# Response shift after coronary revascularization

**DOI:** 10.1007/s11136-021-02902-5

**Published:** 2021-06-22

**Authors:** Tom H. Oreel, Pythia T. Nieuwkerk, Iris D. Hartog, Justine E. Netjes, Alexander B. A. Vonk, Jorrit Lemkes, Hanneke W. M. van Laarhoven, Michael Scherer-Rath, José P. S. Henriques, Frans J. Oort, Mirjam A. G. Sprangers, Mathilde G. E. Verdam

**Affiliations:** 1 Department of Medical Psychology, Amsterdam University Medical Centers, Amsterdam Public Health Research Institute, University of Amsterdam, Amsterdam, Netherlands; 2 Faculty of Philosophy, Theology and Religious Studies, Radboud University Nijmegen, Nijmegen, Netherlands; 3 Department of Cardio-Thoracic Surgery, Amsterdam University Medical Centers, VU University Amsterdam, Amsterdam, Netherlands; 4 Department of Cardiology, Amsterdam University Medical Centers, VU University Amsterdam, Amsterdam, Netherlands; 5 Department of Medical Oncology, Cancer Center Amsterdam, University of Amsterdam, Amsterdam, Netherlands; 6 Department of Cardiology, Amsterdam University Medical Centers, University of Amsterdam, Amsterdam, Netherlands; 7 Research Institute of Child Development and Education, University of Amsterdam, 15776, 1001 NG Amsterdam, Netherlands; 8 Department of Methodology and Statistics, Institute of Psychology, Leiden University, Leiden, Netherlands

**Keywords:** Response shift, Generic health-related quality of life, Disease-specific health-related quality of life, Cardiac disease, Structural equation modelling, Longitudinal data

## Abstract

**Purpose:**

The aims of this study were to investigate (1) the extent to which response shift occurs among patients with coronary artery disease (CAD) after coronary revascularization, (2) whether the assessment of changes in health-related quality of life (HRQoL), controlled for response shift, yield more valid estimates of changes in HRQoL, as indicated by stronger associations with criterion measures of change, than without controlling for response shift, and (3) if occurrences of response shift are related to patient characteristics.

**Methods:**

Patients with CAD completed the SF-36 and the Seattle Angina Questionnaire (SAQ7) at baseline and 3 months after coronary revascularization. Sociodemographic, clinical and psychosocial variables were measured with the patient version of the New York Heart Association-class, Subjective Significance Questionnaire, Reconstruction of Life Events Questionnaire (RE-LIFE), and HEXACO personality inventory. Oort’s Structural Equation Modeling (SEM) approach was used to investigate response shift.

**Results:**

191 patient completed questionnaires at baseline and at 3 months after treatment. The SF-36 showed recalibration and reprioritization response shift and the SAQ7 reconceptualization response shift. Controlling for these response shift effects did not result in more valid estimates of change. One significant association was found between reprioritization response shift and complete integration of having CAD into their life story, as indicated by the RE-LIFE.

**Conclusion:**

Results indicate response shift in HRQoL following coronary revascularization. While we did not find an impact of response shift on the estimates of change, the SEM approach provides a more comprehensive insight into the different types of change in HRQoL following coronary revascularization.

**Supplementary Information:**

The online version contains supplementary material available at 10.1007/s11136-021-02902-5.

## Plain English summary

### What is the key problem/issue/question this manuscript addresses?

To investigate the occurrence of response shift in measures of HRQoL after coronary revascularization and whether the assessment of change in HRQoL, controlled for response shift, yield more valid estimates of changes in HRQoL.

### Why is this study needed?

Response shift effects may influence patients’ evaluations of HRQoL over time and they may bias comparisons of HRQoL data over time. The effectiveness of coronary revascularization on HRQoL is therefore more sensitively assessed when these response shift effects are taken into account.

### What is the main point of your study?

Investigate the occurrence of response shifts in the assessment of change in HRQoL following coronary revascularization using Oort’s Structural Equation Modeling approach.

### Provide a brief overview of your results and what they mean

Results indicate response shift in HRQoL following coronary revascularization. While we did not find an impact of response shift on the estimates of change, the study provides a more comprehensive insight into the different types of change in HRQoL following coronary revascularization. Such research is expected to improve the assessment of change in HRQoL and to obtain a more detailed account of that change.

## Introduction

Patients with coronary artery disease (CAD) typically experience a number of symptoms, such as shortness of breath, chest pain, and fatigue, which impact their daily functioning and health-related quality of life (HRQoL) [1]. The symptoms can become so severe that they require a coronary revascularization procedure to alleviate these symptoms and to improve their HRQoL [2, 3]. Such procedures typically consist of percutaneous coronary intervention (PCI) or coronary artery bypass graft (CABG). Previous research has shown that patients with CAD who undergo revascularization reported better health and HRQoL than patients who did not undergo revascularization [4]. Depending on the type of procedure, patients’ health will generally improve, either abruptly (PCI) or more gradually (CABG) [3, 5–7].

Effectiveness of coronary revascularization procedures on HRQoL is usually measured by comparing scores on HRQoL measures before and after the intervention. However, response shift may influence patients’ evaluations of HRQoL over time. Response shift can be defined as a change in the meaning of patients’ self-evaluation due to a change in internal standards (recalibration), a change in the relative importance of the components of HRQoL (reprioritization), and/or a change in the meaning of the target construct (reconceptualization) [8]. Although response shift can be considered a result of an adaptive process, they may bias comparisons of HRQoL data over time [9, 10]. Changes in HRQoL are therefore more sensitively assessed when these response shift effects are taken into account [9, 10].

The occurrence of response shift in HRQoL data can be investigated using structural equation modelling (SEM) [11]. In this study we investigated response shift in a group of patients with CAD who had at least one comorbidity and who underwent either CABG or PCI, using SEM. The aim of this study was to investigate the extent to which response shift occurs in the measurement of HRQoL of CAD patients after coronary revascularization. We further examined whether the assessment of changes in HRQoL, controlled for response shift effects, yield better change estimates, as indicated by stronger associations with criterion measures of change, than the assessment of changes in HRQoL not controlled for response shift effects. Finally, we investigated whether possible response shift effects are related to patient characteristics, i.e., sociodemographic, clinical and/or psychosocial variables.

We expected that response shift occurs in the measurement of HRQoL of CAD patients undergoing CABG or PCI, because the positive changes in health after coronary revascularization [5, 12] may prompt changes in patients’ views of their HRQoL over time. We expected the associations of HRQoL measures with criterion measures of change to be stronger when response shift is taken into account. With respect to the relations with demographic variables, we expected *age* to be related to response shift, where older patients will show more response shift than younger patients, as they may adapt easier to changes in their HRQoL based on previous exposure to ups and downs in life [13]. We also expected that *gender* is related to response shift, where female CAD patients will show more response shift than male CAD patients, as they have previously been found to manage their physical disability better than male patients [14]. With respect to the clinical variables, we expected that *intervention type* is associated with response shift, with CABG patients showing more response shift than PCI patients, as CABG is a more demanding procedure which evokes greater changes in health [5]. Furthermore, we explored the association between the *number of comorbidities*, for which we did not have a specific hypothesis. With respect to the psychosocial variables, we chose aspects of adaptation and personality that were expected to be associated with response shift. Specifically, we expected a positive association between response shift and *narrative integration:* the extent to which getting CAD is integrated in the life narrative of the patient [15]. We expected that patients who indicated narrative integration are also better able to adapt to positive health changes induced by coronary revascularization [15]. We expected a negative association between *emotionality* and response shift. Patients with high emotionality experience more fear and anxiety [16] and are expected to be less inclined to adapt to the health changes induced by coronary revascularization than patients with low emotionality [17]. Finally, we expected a positive association between *agreeableness* and response shift. Highly agreeable patients are more flexible [18] and are therefore expected to be more inclined to adapt to the health changes induced by coronary revascularization than less agreeable patients [19].

## Methods

### Patients

Patients were recruited at the cardiology departments of the Amsterdam UMC at both Academic Medical Center (AMC) and VU Medical Center (VUmc) locations. All patients were planned for coronary revascularization procedures. They were eligible if they were 18 years or older, had CAD and were scheduled for CABG or PCI. Patients had to have at least one somatic comorbidity. For a complete list of the comorbidities and the exclusion criteria, see Supplementary Material 1. As the local Ethics Committee decided that the Medical Research Involving Human Subjects Act did not apply, the study was exempted from further ethical assessment. Written informed consent was obtained from all patients.

### Outcome variables

#### Generic HRQoL

*Generic HRQoL* was measured with the Dutch version of the Short Form Health Survey 36 (SF-36) [20]. It is a 36-item self-report questionnaire, evaluating physical and mental functioning during the past week using eight subscales: Physical Functioning (PF; 10 items), Role Physical (RP; 4 items), Bodily Pain (BP; 2 items), General Health (GH; 5 items), Mental Health (MH; 5 items), Vitality (VT; 4 items), Social Functioning (SF; 2 items), and Role Emotional (RE; 3 items), with higher scores indicating better functioning (see Supplementary Table 1 for an overview of the items). Subscales scores were calculated by the weighted sum scores of their associated item scores and transformed into a 0–100 scale, with higher scores indicating better HRQoL. Component scores for physical health (PCS) and mental health (MCS) were calculated by combining the associated subscale scores and transforming them into a 0–100 scale, with higher scores indicating better physical and mental functioning, respectively.

#### Disease-specific HRQoL

*Disease-specific HRQoL* was measured with the short version of the Seattle Angina Questionnaire (SAQ7) [21]. The SAQ7 is a self-report 7-item questionnaire, which evaluates the impact of CAD on three domains: Physical Functioning (3 items), Angina Frequency (2 items), and Quality of Life (2 items) (see Supplementary Table 1 for the questionnaire items). Item scores were linearly converted to a 0–100 scale, with higher scores indicating better disease-specific HRQoL (i.e., less physical limitations, less angina symptoms, and better quality of life). Scale scores were calculated by taking the mean of the associated item scores, with higher scores indicating better disease-specific HRQoL.

#### Criterion measures of change

*Subjective change in HRQoL* was measured with the Subjective Significance Questionnaire (SSQ) [22]. The six-item SSQ measures subjective change since revascularization on: Overall Quality of Life, Fatigue, Physical Condition, Pain, Social Activities, and Emotional State. Items were rated on a 7-point scale. Scores ranging from 1 to 3 and 5 to 7 indicate a decline and improvement, respectively, with lower scores indicating more decline and higher scores more improvement in HRQoL since the coronary revascularization procedure. An item-score of 4 indicates no change in HRQoL.

*Functional limitations* due to CAD symptoms were measured with the patient version of the New York Heart Association (*NYHA*) functioning classification system [23, 24]. Respondents had to choose one of the following options: I = “not limited in physical activities”, II = “somewhat limited in physical activities”, III = “fairly limited in physical activities”, IV = “not capable of physical activities”. *Change in NYHA class* (subtraction of the assessment after from the one before the revascularization) ranges from − 3 to + 3, with positive change scores indicating improved physical functioning.

### Patient characteristics

#### Sociodemographic and clinical variables

Patients reported their *age* (in years) and *gender* (“male” or “female”). *Intervention type* (PCI or CABG) and *number of comorbidity conditions* (number of identified conditions ≤ 1 or ≥ 2) were identified using the hospitals’ electronic medical records. If records were unclear, they were checked by two medical specialists (JH and HvL).

#### Psychosocial variables

*Narrative integration* refers to the extent to which the heart condition is given a meaningful place in patients’ life story. Narrative integration was assessed using the Reconstruction of Life Events Questionnaire (RE-LIFE) [15]. We used the 3-item subscale *receiving*, which indicates complete integration of the event (getting CAD) into the life story, embracing the positive new possibilities that emerge from the life event*.* Items were rated on a 5-point scale. Scale scores were calculated as mean item scores, with higher scores indicating more narrative integration (Hartog et al., submitted). *Emotionality and Agreeableness* were assessed with the HEXACO Personality Inventory—Dutch, simplified version (HEXACO-SPI) [25]. Both personality dimensions are assessed with 16 items rated on a 5-point scale. Scale scores for both dimensions were calculated as mean item scores, with higher scores indicating more emotionality and more agreeableness, respectively.

### Procedure

Questionnaires on HRQoL (generic and disease-specific) and the NYHA class were completed at four time-points: at 1 week prior to revascularization (baseline), and 2 weeks, 3 months and 6 months after revascularization (follow-up). Sociodemographic and clinical information was collected at baseline. SSQ, RE-LIFE, and HEXACO-SPI were assessed at the 3-month follow-up. Patients could choose to complete the questionnaires on paper or online. For this study we only used baseline and 3 months follow-up data as we expected response shift effects to be most prevalent at that time.

### Statistical analysis

Oort’s [11] SEM procedure was used to investigate response shift, where the associations between observed scores (i.e., scores of the SF-36 and SAQ7 questionnaires) and the underlying construct of interest (generic and disease-specific HRQoL) are specified with a factor model. By fitting a factor model to the data from both baseline and follow-up assessments, change in model parameters can be used to distinguish response shift effects from change in the underlying construct (HRQoL). For both the SF-36 and the SAQ7, the SEM procedure was conducted in six steps: (1) establishing a measurement model, (2) overall test of response shift, (3) detection of specific response shift effects, (4) investigation of the impact of response shift on the assessment of change in HRQoL, (5) assessment of criterion validity of change in HRQoL, and (6) prediction of detected response shift effects; see a detailed description of the six steps below. The six steps were used to test the three objectives: (1) investigate the extent to which response shift occurs in HRQoL (steps 1–3), (2) examine whether changes in HRQoL, controlled for response shift effects, yield more valid estimates of change as compared to changes in HRQoL that are not controlled for response shift effects (steps 4 and 5), and (3) investigate associations between patient characteristics and the occurrence of response shift (step 6). Statistical analyses were performed using the R-package Lavaan version 0.6–5 [26]. We used Maximum Likelihood (ML) estimation as the data that were analyzed consisted of at least five response categories, and were therefore considered to sufficiently approximate continuous interval scales. ML estimation yields a chi-square test statistic that can be used to evaluate overall fit of the model and differences in model fit, and provides ML estimates of all model parameters that can be used to evaluate the statistical significance of individual parameters in the model.

#### Step 1: establishing a measurement model

In the first step of the SEM procedure we established an interpretable and well-fitting “measurement model”. The measurement model specifies the relationships between the observed scores (i.e., SF-36 scale scores and SAQ item scores) and the underlying latent variables (i.e., generic and disease-specific HRQoL. Goodness-of-fit of the measurement model was evaluated with the *Chi-square test of exact fit* (chi-square test), where a non-significant chi-square test (alpha = 0.05) indicates an exact fit between the factor model and the data. However, because in practice exact fit between the model and the data are rare, we also looked at several indices of approximate fit, namely the *Root Mean Square Error of Approximation* (RMSEA; [27]) the *Comparative Fit Index* (CFI; [28]) and the *Standardized Root Mean Square Residual* (SRMR; [29]). An RMSEA value below 0.08 indicates ‘reasonable’ fit and an RMSEA below 0.05 ‘good’ fit [27, 30]. A CFI between 0.95 and 0.97 is ‘reasonable’ fit, a CFI of 0.97 or higher is indicative of ‘good’ fit. A SRMR of 0.08 or less is indicative of ‘reasonable’ fit, and an SRMR below 0.05 ‘good’ fit [30]. As a rule of thumb, the overall model fit of the measurement model was considered to be sufficient when at least two out of three approximate fit indices indicated at least ‘reasonable’ fit (see [31] for a discussion on the complexities of model fit evaluation). When the model fit of the measurement model was not sufficient, we modified the measurement model until we arrived at a well-fitting and interpretable model. To inspect possible indications of model misfit we used modification indices [32], and each modification to the measurement model was tested using the difference in chi-square values of both models with the *chi-square difference test* (∆*χ*^2^), where a significant chi-square (alpha = 0.05) indicates that the modification significantly improves model fit.

#### Step 2: overall test of response shift

The second step of the SEM procedure was to fit a model of no response shift in which all parameters associated with response shift (i.e., factor loadings and intercepts) were constrained to be equal across time. To test for the presence of response shift, the model fit of the “no response shift model” was compared to the model fit of the measurement model using the *chi-square difference test* (∆*χ*^2^). A significant deterioration in model fit (alpha = 0.05) indicates the overall presence of response shift (any type of response shift).

#### Step 3: detection of specific response shift effects

We then investigated which variables [i.e., scale score (SF-36) or item-score (SAQ7)] were affected by which type of response shift. We used an iterative procedure in which the presence of each type of response shift was investigated for each variable, one by one. Response shift was operationalized as across-measurement differences in model parameters, where a change in factor structure is indicative of *reconceptualization*, a change in the value of factor loadings is indicative of *reprioritization*, and a change in the intercepts is indicative of uniform *recalibration* [11]. In the first iteration, we started with the no response shift model and included each model parameter associated with response shift one at a time, and tested whether the improvement in fit, as compared to the no response shift model, was significant (alpha = 0.05). For each iteration, we tested the statistical significance for all possible response shift effects. The model parameter that led to the largest improvement in model fit (indicated by the ∆*χ*^2^) was included in the model. With each modification we also considered whether inclusion of the response shift effect could be justified theoretically. If different response shift effects yielded similar improvement in model fit, we decided on which response shift effect to include in the model based on substantive considerations. This iterative process was repeated until we arrived at a model which did not differ in model fit compared to the measurement model (as indicated by a non-significant ∆*χ*^2^ with alpha = 0.05) and/or no further indications of statistically significant or sensible model modifications were found. The final model that includes all indications of response shift is referred to as the “response shift model”. Effect sizes (Cohens *d*) of the detected response shift effects were calculated using parameter estimates of the final model [33], where values of 0.20, 0.50, and 0.80 can be interpreted as indicating ‘small’, ‘moderate, and ‘large’ effects, respectively [34].

#### Step 4: impact of response shift on the assessment of change in HRQoL

We subsequently assessed the impact of response shift on the assessment of change in (general and disease specific) HRQoL. To investigate the impact of response shift on the change in HRQoL, we compared the estimates of change of the underlying latent factors between the no response shift model (i.e., without taking into account response shift) and the response shift model, where response shifts are taken into account. To provide further reference for the interpretation of change in HRQL, we also compared the estimated change in the underlying latent factors with the observed change, that is, the change in observed scores (scores of the SF-36 and SAQ7 questionnaires). Effect sizes (Cohens *d*) of the observed change scores and the latent change scores were calculated using the standardized response mean, where values of 0.2, 0.5 and 0.8 were considered ‘small’, ‘moderate’ and ‘large’ effects, respectively [34].

#### Step 5: criterion validity

We correlated the change in the underlying latent factors of general and disease-specific HRQoL with the criterion variables of change in HRQoL, that is change in NYHA class and SSQ. To correctly capture the mean change and individual variability in change of general and disease-specific HRQL, we used latent change score (LCS) models [35, 36]. The LCS model is merely a re-specification of the SEM model that was used for response shift investigation, and thus does not alter the results. To investigate the impact of response shift on the criterion validity of change in HRQoL, we compared the correlations of the LCS factors for general and disease-specific HRQoL with the criterion variables of change between the no response shift model and the response shift model. Furthermore, we also calculated the correlations (Pearson) between the change in observed scores (SF36 and SAQ7) with the criterion variables of change in HRQoL.

#### Step 6: associations of patient characteristics with detected response shift

We extended the final response shift model to investigate possible associations between patient characteristics (i.e., sociodemographic, clinical and/or psychosocial variables) and the detected response shift effects. The model included direct effects of the patient characteristics on the scale(s) or item(s) for which response shift was detected. When an effect a patient characteristics on an item is significant (alpha = 0.05), this indicates that the patient characteristics is associated with the detected response shift. Standardized effects of the associations between patient characteristics and response shift can be interpreted as correlation coefficients, where values of 0.1, 0.3 and 0.5 are indicative of ‘small’, ‘moderate’, and ‘large’ effects, respectively [34].

## Results

### Patients

A total of 467 patients were approached for the study, of whom 144 patients declined participation (31%), and 3 patients were excluded because their coronary revascularization was delayed. Of the remaining 320 patients, 67 did not undergo coronary revascularization but a diagnostic procedure instead (e.g., coronary angiography), seven patients missed the baseline and 55 patients missed the follow-up assessment, resulting in a final sample size of 191. Background variables are shown in Table 1.

**Table 1:** Background variables

Background variables	*N*
Total number of patients	191
Male patients (%)	139 (73)
Age (in years)	
Mean (SD)	68.42 (8.67)
Median	69
Range	46–86
Number of comorbidities	
1 (%)	75 (39)
2 or more (%)	116 (61)
Type of Intervention	
PCI (%)	145 (76)
CABG (%)	46 (24)

*PCI* percutaneous coronary intervention, *CABG* coronary artery bypass graft

### Generic HRQoL

The means and standard deviations of the SF-36 subscales and component scores at baseline and follow-up are provided in Table 2. In general, there is a mean improvement for all scores over time. Skewness and Kurtosis statistics provided in Table 2 are all in the normal range of < 3 and < 10, respectively [37], indicating that all scores are normality distributed.

**Table 2:** Means and standard deviations of the SF-36 scales and component scores, SAQ7 items and scale scores, and the SSQ and NYHA class criterion measures of change at baseline and follow-up, including skewness and kurtosis statistics

	Baseline			Follow-up		
Scales/items	Mean (SD)	Skewness	Kurtosis	Mean (SD)	Skewness	Kurtosis
SF-36						
PCS	36.02 (9.96)	0.13	− 0.70	41.66 (10.17)	− 0.35	− 0.57
PF	51.03 (25.28)	− 0.05	− 0.93	64.81 (25.84)	− 0.63	− 0.58
RP	28.85 (39.32)	0.90	− 0.88	54.32 (42.99)	− 0.16	− 1.72
RE	55.65 (43.74)	− 0.15	− 1.73	70.20 (40.70)	− 0.75	− 1.18
VT	51.40 (20.46)	− 0.17	− 0.47	59.81 (20.99)	− 0.28	− 0.53
MCS	47.65 (11.22)	− 0.12	− 0.92	51.09 (10.96)	− 0.90	0.06
MH	68.54 (19.34)	− 0.31	− 0.62	75.02 (20.28)	− 0.87	0.14
SF	67.80 (25.47)	− 0.52	− 0.39	77.73 (22.33)	− 0.81	− 0.16
BP	63.30 (23.51)	− 0.06	− 0.85	71.47 (24.07)	− 0.37	− 0.99
GH	48.06 (18.31)	0.21	− 0.65	55.92 (20.12)	0.07	− 0.52
SAQ7						
PF	60.31 (36.23)	− 0.25	− 1.13	74.15 (31.45)	− 0.93	0.06
PF 1	82.73 (23.66)	− 1.34	1.01	89.44 (20.97)	− 2.41	6.37
PF 2	55.18 (33.89)	− 0.23	− 1.30	71.24 (30.32)	− 0.96	− 0.03
PF 3	39.78 (35.71)	0.36	− 1.27	56.34 (34.91)	− 0.32	− 1.23
AF	79.59 (27.64)	− 0.94	0.17	93.32 (17.41)	− 2.57	8.09
AF 1	68.94 (30.36)	− 0.67	− 0.70	88.13 (21.75)	− 2.27	5.13
AF 2	89.21 (20.68)	− 1.97	3.01	97.07 (11.54)	− 5.62	36.42
QL	47.82 (31.24)	0.07	− 0.92	74.93 (25.32)	− 0.82	− 0.32
QL 1	57.98 (30.00	− 0.32	− 0.93	79.39 (24.29)	− 1.11	0.69
QL 2	38.19 (28.33)	0.40	− 0.74	69.25 (26.84)	− 0.83	0.07
SSQ						
Overall QoL				5.15 (1.36)	− 0.55	− 0.45
Fatigue				4.80 (1.40)	− 0.39	− 0.36
Physical Condition				4.78 (1.43)	− 0.38	− 0.58
Pain				5.49 (1.47)	− 0.61	− 0.78
Social Activities				4.72 (1.43)	0.16	− 0.72
Emotional Sate				4.56 (1.31)	0.31	− 0.84
Change NYHA class				0.55 (0.89)	0.25	0.53

*PCS* physical component, *PF (SF-36)*  Physical Functioning, *RP* Role Physical, *RE* Role Emotional, *VT* Vitality, *MCS* mental component, *MH* Mental Health, *SF* Social Functioning, *BP* Bodily Pain, *GH* General Health, *PF* 1,2,3 (SAQ7)  Physical Functioning items, *AF* 1,2 Angina Frequency items, *QL* 1,2 Quality of Life items, *SSQ* Subjective Significance Questionnaire [22], *QoL* subjective change in Overall Quality of Life, *Change* *NYHA* change in the New York Heart Association functioning classification system [23, 24]

#### The occurrence of response shift (steps 1–3)

##### Step 1

**Fig. 1:**
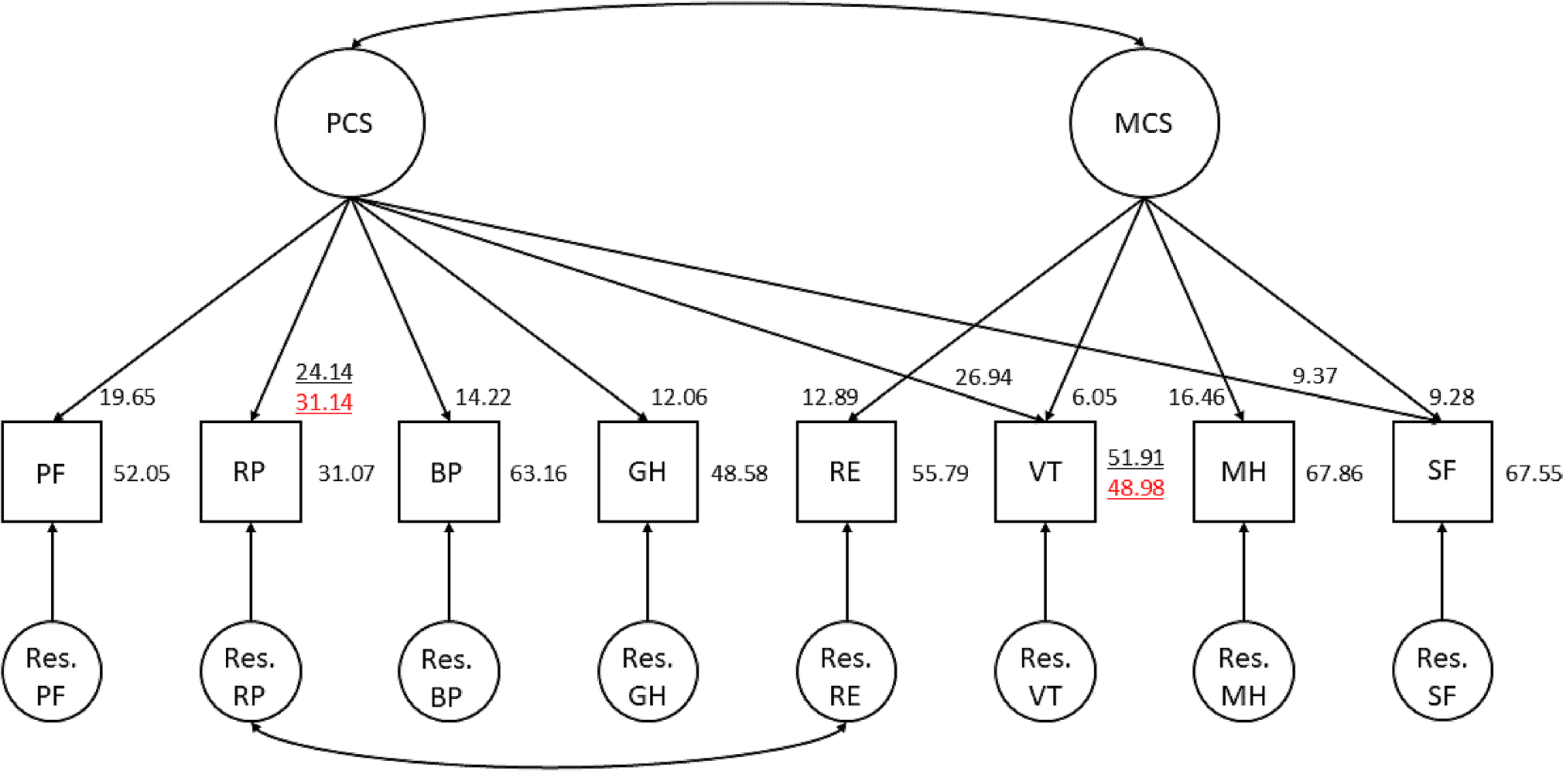
Measurement model of the SF-36 used in the response shift analyses. The numbers represent the maximum likelihood estimates of the model parameters associated with response shift, i.e., factor loadings and intercepts. The underlined numbers represent the maximum likelihood estimates of the model parameters associated with the detected recalibration and reprioritization response shift (i.e., intercepts and factor loadings, respectively). The underlined black number represents the parameter estimate at baseline. The underlined red number represents the parameter estimate at follow-up. All other parameter estimates were constrained to be equal across measurements. *PCS* physical component, *MCS* mental component, *PF* Physical Functioning, *RP* Role Physical, *BP* Bodily Pain, *GH* General Health, *RE* Role Emotional,* VT* Vitality, *MH* Mental Health, *SF* Social Functioning

Based on the manual of the SF-36 [20] we chose a two-factor model as a starting point for the measurement model (see Fig. 1). The initial measurement model showed poor overall model fit (see Table 3, step 1). To arrive at a well-fitting measurement model we included three model modifications, including the addition of a residual covariance between item RP and RE, a factor loading of VT on the factor PCS, and a factor loading of SF on the factor PCS. With these modifications the measurement model showed good (CFI) and reasonable (RMSEA and SRMR) overall model fit and was considered the final measurement model (see Table 3; Fig. 1). Technical details and theoretical justifications of step 1 are provided in Supplementary Material 2.

**Table 3:** Goodness of overall model fit for the models used in steps 1–3 of the SEM procedure for the investigation of response shift in the SF-36

Steps	*χ*^2^ (df)	CFI	RMSEA [90% CI]	SRMR
Step 1: Measurement model				
Initial measurement model	283.082 (90)	0.893	0.109 [0.095–0.123]	0.061
+ Residual covariance RP-RE	244.305 (86)	0.912	0.101 [0.086–0.116]	0.059
+ Factor loading VT on PCS	207.743 (84)	0.931	0.090 [0.075–0.106]	0.061
+ Factor loading SF on PCS (final measurement model)	176.174 (82)	0.948	0.080 [0.063–0.096]	0.058
Step 2: No response shift model	202.068 (96)	0.941	0.078 [0.063–0.093]	0.067
Step 3: Response shift model				
+ Reprioritization RP	195.994 (95)	0.944	0.077 [0.062–0.091]	0.061
+ Recalibration VT (final response shift model)	190.277 (94)	0.947	0.075 [0.060–0.091]	0.062

*Df* degrees of freedom, *CFI* Comparative Fit Index, *RMSEA* Root Mean Square Error of Approximation, *CI* confidence interval, *SRMR* Standardized Root Mean Square Residual, *RP* Role Physical, *RE* Role Emotional, *VT* Vitality, *PCS* physical component, *SF* Social Functioning

##### Step 2

The model that assumed no response shift (i.e., the no response shift model) fitted significantly worse as compared to the measurement model (∆*χ*^2^ = 25.894 (14), *p* = 0.027), indicating the overall presence of response shift (see Table 3, step 2 and Supplementary Material 2).

##### Step 3

Two cases of response shift were identified (see Fig. 1). The fit of the response shift model that included these two response shifts was good (see Table 3, step 3) and showed equivalent model fit as compared to the measurement model (∆*χ*^2^ (12) = 14.102, *p* = 0.294). First, a change in the factor loading of RP on PCS was identified (∆*χ*^2^ (1) = 6.07, *p* = 0.014), indicating reprioritization for RP. Inspection of parameter estimates showed that the factor loading of RP on PCS was larger at follow-up, indicating that RP became more ‘important’ to the measurement of physical health (Cohen’s *d* = 0.12). Second, a change in the intercept of VT was identified (∆*χ*^2^ (1) = 6.23, *p* = 0.013), indicating recalibration of VT. Inspection of the model parameters showed that the intercept of VT was smaller at follow-up, indicating that the measurement scale of VT may have shifted such that it was ‘easier’ to score lower on VT at follow-up as compared to baseline (Cohen’s *d* = − 0.14).

#### Impact of response shift on the assessment of change (steps 4 and 5)

##### Step 4

Effect sizes of change in generic HRQoL using the SEM procedure were larger (0.45–0.87) than those for observed change scores (0.32–0.62) and were more precise as indicated by smaller confidence intervals (see Table 4). Comparing the estimates of change between the no response shift model and the response shift model indicated that the occurrence of response shift did only marginally impact the estimated improvement in physical (0.87 versus 0.83) and mental health (0.47 versus 0.45).

**Table 4:** Effect sizes (ES) of change (standardized response mean) and confidence intervals (CI) for both the physical (PCS) and mental (MCS) components of general HRQoL as measured with the SF-36

	Observed change SE [95% CI]	Change no response shift model^a^ SE [95% CI]	Change response shift model^a^ SE [95% CI]
PCS	0.62 [0.32–0.91]	0.83 [0.72–0.94]	0.87 [0.76–0.98]
MCS	0.32 [0.02–0.61]	0.45 [0.33–0.56]	0.47 [0.35–0.59]

^a^Reported change scores are the estimated changes in the latent factor means from the associated statistical models

##### Step 5

In general, the criterion measures were more strongly related to changes in PCS than to changes in MCS (see Table 5). Furthermore, the estimated change in the SEM method correlated more strongly with the criterion measures than the observed change scores. The correlations of the criterion measures with changes in PCS and MCS in the no response shift model were identical to those in the response shift model, with the exception of the correlation with SSQ emotional state (Table 5). Taking response shift into account did not increase the criterion validity of changes in HRQoL.

**Table 5:** Correlations between the change scores of the physical (PCS) and mental (MCS) components of general HRQoL with the criterion measures of change in HRQoL

Criterion variables	Observed change		No response shift model^a^		Response shift model^a^	
	PCS	MCS	PCS	MCS	PCS	MCS
Change NYHA class	0.48	0.17	0.60	0.38	0.60	0.38
SSQ						
Overall QoL	0.39	0.14	0.61	0.39	0.61	0.39
Fatigue	0.42	0.19	0.70	0.43	0.70	0.43
Physical Condition	0.43	0.22	0.74	0.50	0.74	0.50
Pain	0.32	0.09	0.45	0.28	0.45	0.28
Social Activities	0.35	0.19	0.67	0.41	0.67	0.41
Emotional Sate	0.26	0.23	0.54	0.56	0.56	0.53
Mean correlation	0.38	0.18	0.60	0.42	0.62	0.42

*Change NYHA* change in the New York heart association functioning classification system [23, 24], *SSQ* Subjective Significance Questionnaire [22]

^a^PCS and MCS here refer to the latent factors from the associated statistical models

#### Associations of patient characteristics with detected response shift (step 6)

##### Step 6

We examined the associations of sociodemographic, psychosocial and clinical variables with the detected response shift effects. We found one significant association between reprioritization of RP and *receiving* (*r* = 0.12, *p* = 0.029), indicating that patients who scored high on receiving (indicating complete integration of having CAD into their life story) were especially prone to show reprioritization response shift of RP. We found no significant associations with the detected recalibration response shift of VT.

### Disease-specific HRQoL

The means and standard deviations of the SAQ7 items at baseline and follow-up are provided in Table 2. In general, there is a mean improvement for all scores over time. All skewness and Kurtosis statistics provided in Table 2 are in the normal range of < 3 and < 10, respectively [37], except for the second item of angina frequency at follow-up. We continued our analyses as planned as the deviation from normality is only present in one item-score and ML estimation is robust against moderate violations of normality [38].

#### The occurrence of response shift (steps 1–3)

##### Step 1

**Fig. 2:**
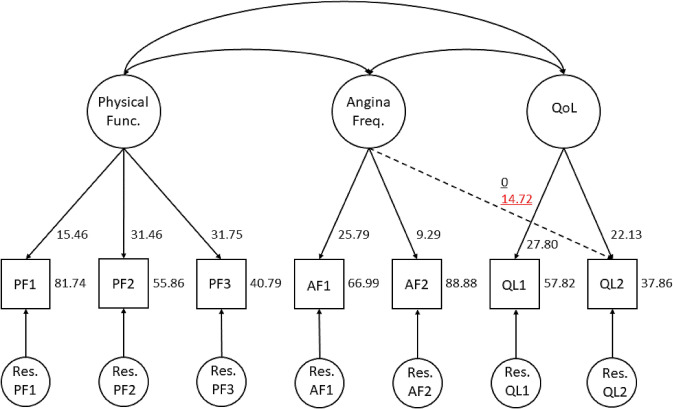
Measurement model of the SAQ7 used in the response shift analyses. The numbers represent the maximum likelihood estimates of the model parameters associated with response shift, i.e. factor loadings and intercepts. The dotted line represents reconceptualization of QL2 and the underlined numbers represent the maximum likelihood estimate of the model parameters associated with reconceptualization, i.e. factor loadings. The underlined black number represents the parameter estimate at baseline. The underlined red number represents the parameter estimate at follow-up. All other parameter estimates were constrained to be equal across measurements. *PF 1,2,3* Physical Functioning items, *AF 1,2* Angina Frequency items, *QL 1,2* Quality of Life items

Based on the three components of the SAQ7 [21], we started with a three-factor model, including Physical Functioning (PF1, PF2, PF3), Angina Frequency (AF1 and AF2), and Quality of Life (QL1 and QL2) (see Fig. 2). Since the initial measurement model showed acceptable, we considered this to be the final measurement model (see Table 6 step 1).

**Table 6:** Goodness-of-fit of overall model fit for the models used in steps 1–3 of the SEM procedure for the investigation of response shift in the SAQ7 data

Steps	χ ^2^ (df)	CFI	RMSEA [90% CI]	SRMR
Step 1: Measurement model	94.239 (55)	0.962	0.070 [0.045–0.093]	0.053
Step 2: No response shift model	111.374 (63)	0.953	0.073[0.050–0.094]	0.073
Step 3: Response shift model				
+ Reconceptualization QL2 (final response shift model)	110.487 (62)	0.969	0.059[0.033–0.083]	0.063

*QL 2* Quality of Life item 2, *df* degrees of freedom, *CFI* Comparative Fit Index, *RMSEA* Root Mean Square Error of Approximation, *CI* confidence interval, *SRMR* Standardized Root Mean Square Residual

##### Step 2

The no response shift model fitted significantly worse than the measurement model (∆χ^2^ (8) = 17.136, *p* = 0.029), indicating the presence of response shift effects (see Table 6 step 2).

##### Step 3

One case of response shift was identified (see Fig. 2). The resulting response shift model showed good overall fit, and comparable fit to the measurement model (see Table 6, step 3). A factor loading of QL2 on Angina Frequency at follow-up was added, indicating reconceptualization of QL2. After treatment, Angina Frequency became directly associated with patients’ scores on item QL2 that asks about the outlook on quality of life given the current CAD symptoms (that is, more strongly associated with the scores on this item than with the scores on item QL1 that asks about current quality of life given the CAD symptoms) (Cohen’s *d* = 0.46).

#### Impact of response shift on the assessment of change (steps 4 and 5)

##### Step 4

Effect sizes of change in disease-specific HRQoL using the SEM procedure (0.63–1.08) were larger than those for the observed change scores (0.55–0.95) and were more precise as indicated by the smaller confidence intervals (see Table 7). Taking into account the occurrence of response shift resulted in lower estimated improvement in Quality of Life (0.83 versus 1.08), did only marginally impact the improvement in Angina Frequency (0.88 versus 0.89) and did not impact the improvement in Physical Functioning (both 0.63).

**Table 7:** Effect sizes (ES) of change (standardized response mean) and confidence intervals (CI) for Physical Functioning, Angina Frequency and Quality of Life of disease-specific HRQoL as measured with the SAQ7

	Observed change SE[95% CI]	Change no response shift model^a^ SE[95% CI]	Change response shift model^a^ SE[95% CI]
Physical Functioning	0.55 [0.26–0.85]	0.63 [0.52–0.74]	0.63 [0.52–0.74]
Angina Frequency	0.62 [0.32–0.91]	0.89 [0.76–1.02]	0.88 [0.76–1.01]
Quality of Life	0.95 [0.61–1.25]	1.08 [0.94–1.22]	0.83 [0.70–0.97]

^a^Reported change scores are the estimated changes in the latent factor means from the associated statistical models

##### Step 5

In general, the criterion measures were most strongly related to Quality of Life, followed by Angina Frequency and Physical Functioning, respectively (see Table 8). Moreover, the estimated change scores of the SEM method correlated more strongly with the criterion measures than the observed change scores. The correlations of the no response shift model and the response shift model were almost identical. Taking response shift into account did not increase the criterion validity of changes in HRQoL.

**Table 8:** Correlations between the change scores of the Physical Functioning (PF), Angina Frequency (AF) and Quality of Life (QoL) of disease-specific HRQoL with the criterion measures of change in HRQoL

Criterion variables	Observed change			No response shift model^a^			Response shift model^a^		
	PF	AF	QoL	PF	AF	QoL	PF	AF	QoL
Change NYHA class	0.39	0.31	0.54	0.26	0.45	0.58	0.28	0.45	0.58
SSQ									
Overall QoL	0.17	0.26	0.30	0.38	0.68	0.63	0.38	0.66	0.58
Fatigue	0.11	0.06	0.31	0.35	0.38	0.66	0.36	0.43	0.65
Physical Condition	0.14	0.01	0.33	0.44	0.37	0.70	0.45	0.41	0.70
Pain	0.18	0.24	0.24	0.36	0.59	0.41	0.36	0.53	0.38
Social Activities	0.19	0.12	0.30	0.41	0.44	0.59	0.41	0.41	0.59
Emotional State	0.04	0.13	0.23	0.23	0.40	0.52	0.23	0.38	0.52
Mean	0.17	0.16	0.32	0.35	0.47	0.58	0.35	0.47	0.57

*Change NYHA class* change in the New York heart association functioning classification system [23, 24], *SSQ* Subjective Significance Questionnaire [22], *PF* Physcial Functioning, *AF* Angina Frequency, *QoL* Quality of Life

^a^PF, AF, and QoL here refer to the latent factors from the associated statistical models

#### Associations of patient characteristics with the detected response shift (step 6)

##### Step 6

We found no significant associations between patient characteristics and the detected reconceptualization response shift.

## Discussion

The present study indicates the occurrence of response shifts in the assessment of HRQoL following coronary revascularization using SEM. In generic HRQoL we found a reprioritization response shift; role limitations due to physical health became more important for their physical health after revascularization. This finding highlights the relatively strong improvement of the RP scale over time and could indicate an adaptive process in which patients learn to navigate their lives with limited physical functioning. We also found a recalibration response shift in generic HRQoL; the internal criteria for the vitality response scale shifted after revascularization, such that it became easier for patients to score lower on vitality. That is, even if patients showed the same generic HRQoL after revascularization as compared to baseline, they did indicate to be less vital. It could be that, shortly after revascularization, patients experienced to be much less vital as compared to baseline, which resulted in a re-interpretation of the response scale. Previous research on response shift in generic HRQoL, using the SF-36, among CAD patients over a 1-year period also found evidence for recalibration albeit in the physical functioning scale [39]. While response shift was found for a different scale, these results are in line with our reprioritization and recalibration findings that CAD patients adapt their evaluations of physical health over time. In disease-specific HRQoL we found one reconceptualization response shift; after revascularization, Angina Frequency became directly associated with how patients rated their outlook on QoL given their CAD symptoms. That is, scores on the item about patients’ outlook on QoL were not only indicative of QoL, but also of Angina Frequency. This indicates that experiencing angina symptoms becomes more important for patients’ outlook on QoL after revascularization. It could be that, during recovery, patients became aware of the importance of angina symptoms on their outlook on QoL, therefore revising their conceptualization of angina symptoms. The importance of angina symptoms for the presence of CAD is well documented. For example, angina was the most important predictor of obstructive CAD [40]. Not surprisingly, angina is also important for patients’ HRQoL. For example, an improvement in angina symptoms was found to be associated with an improvement in patients’ HRQoL after a nurse-led transitional care program for CAD patients [41]. It should be noted that the response shift effects found in our study had only a minor impact on the assessment of change in HRQoL and did not result in better criterion validity of change in HRQoL as we had expected. Finally, only one response shift effect was found to be associated with one patient characteristic.

The question arises why these response shift effects did not improve the measurement of change when taking these effects into account. The response shift effects for the SF-36 were of a small magnitude and moderate for the SAQ7. This is in line with the general finding that median response shift effect sizes are generally small but may vary widely [42]. When the effects of response shift on the assessment of change are relatively minor, it is to be expected that controlling for these response shift effects will not result in significantly better estimates of change in HRQoL.

We only found one significant association between patient characteristics and response shift. Individuals who scored high on the receiving scale were especially prone to show reprioritization response shift on the role physical subscale, an association in the expected direction. For example, it could be that patients who embraced the positive new possibilities that emerge from having CAD are more likely to recognize the importance of their social role during recovery. Contrary to our expectations we did not find any significant relationship between response shift and other patient characteristics. As CAD often affects older males; the relatively small sample size of the current study and its associated skewed distributions of age and gender may have reduced the power of detecting significant associations. We only included patients with at least one comorbidity which may have reduced the chance of finding a significant relationship with the number of comorbidities, as the effect of comorbidity might be strongest between patients with versus without comorbidities. Most surprisingly, we did not find any association between response shift and intervention type. We expected CAD patients undergoing CABG to experience more response shift than patients undergoing PCI, as their health will change to a greater extent. However, CABG patients formed only a quarter of the study population whereby the uneven distribution may have explained the null findings. Finally, we did not find significant associations between the personality dimensions emotionality and agreeableness with response shift. A possible explanation is that these dimensions are too distal for response shift and that for example adaptability and flexibility are more proximal for response shift.

### Limitations and strengths

Some limitations should be noted. First, while our sample of primarily older, white men is representative of this disease population, the homogeneity may have limited the finding of significant effects. Second, most patients received a PCI, for which the impact on patients’ health may have been too small to evoke response shift. Third, most patient characteristics did not explain the detected response shift effects. Other, unmeasured, variables may explain these effects, including disease severity or type of CAD. Therefore, future research is needed to corroborate our findings and possibly extend the type and number of explanatory variables included in the analyses. Moreover, we analyzed data obtained 3 months after revascularization as we expected response shift effects to be most prevalent at that time. However, since the optimal timing for response shift detection is unknown, response shift effects may have occurred later for those patients who were not yet fully recovered from CABG.

This study has also a number of strengths. We carefully recruited eligible consecutive patients from two major cardiology referral centres with medically confirmed diagnoses and comorbidities. We only investigated patients who received either PCI or CABG, as we expected both procedures to induce sufficiently large health changes to prompt response shift effects. We used both the SF-36 and SAQ7, commonly used and valid to assess generic and disease-specific HRQoL, providing a comprehensive picture of HRQoL. We also used well-established and validated questionnaires for both our criterion and patient characteristics (with the exception of narrative integration scale). Importantly, the inclusion of response shift effects was based on both statistical and theoretical considerations. Finally, this study is one of the first to our knowledge to apply Oort’s SEM procedure to test the criterion validity of HRQoL measures controlled for response shift and to test the association between patient characteristics and response shift effects [43].

### Conclusions

This study demonstrates that with the SEM approach it is possible to investigate the three types of response shift effects, account for these response shift effects when assessing change, estimate the criterion validity of HRQoL measures controlled for response shift effects, and investigate associations between patient characteristics and response shift effects. While we did not find an impact of response shift on the estimates of change in HRQoL, our method allows for a more precise measurement of HRQoL and gives comprehensive insight into the different types of change in HRQoL following coronary revascularization. More research is needed, particularly to better understand the relationship between different patient characteristics, clinical variables and response shift effects and their impact on the assessment of change. Such research is expected to improve the assessment of change in HRQoL and to obtain a more detailed account of that change.

## Supplementary Information

Below is the link to the electronic supplementary material.

Supplementary file1 (DOCX 18 KB)

## Data Availability

Available upon request.
