# Supplementary material for: Response shift after coronary revascularization
Source: Qual Life Res. 2021 Jun 22;31(2):437–50. doi: 10.1007/s11136-021-02902-5 (PMC8847301; doi:10.1007/s11136-021-02902-5)
Supplement: Supplementary file 1 — Supplementary file1 (DOCX 18 KB) [file 11136_2021_2902_MOESM1_ESM.docx]

**SUPPLEMENTAL MATERIALS TO:**

**RESPONSE SHIFT AFTER CORONARY REVASCULARIZATION**

Tom H. Oreel, Pythia T. Nieuwkerk, Iris D. Hartog, Justine E. Netjes, Alexander B.A. Vonk, Jorrit Lemkes, Hanneke W.M. van Laarhoven, Michael Scherer-Rath, José P.S. Henriques, Frans J. Oort, Mirjam A.G. Sprangers, Mathilde G.E. Verdam

Corresponding author: [m.g.e.verdam@amsterdamumc.nl](mailto:m.g.e.verdam@amsterdamumc.nl)

**Supplementary Table 1** Overview of the items of the SF-36 and the SAQ7

| **SF36** |
| --- |
| *Mental Health (MH)* |
| Have you been a very nervous person? |
| Have you felt so down in the dumps that nothing could cheer you up? |
| Have you felt calm and peaceful? |
| Have you felt downhearted and blue? |
| Have you been a happy person? |
| *General Health (GH)* |
| In general, would you say your health is…? |
| I seem to get sick a little easier than other people |
| I am as health as anybody I know |
| I expect my health to get worse |
| My health is excellent |
| *Physical functioning (PF)* |
| Vigorous activities |
| Moderate activities |
| Lifting or carrying groceries |
| Climbing several flights of stairs |
| Climbing one flight of stairs |
| Bending, kneeling, or stooping |
| Walking more than a mile |
| Walking several blocks |
| Walking one block |
| Bathing or dressing yourself |
| *Role limitations due to physical health (RP)* |
| Did you cut down on the amount of time you spent on work or other activities? |
| Did you accomplish less than you would like? |
| Were you limited in the kind of work or other activities? |
| Did you have difficulty performing the work or other activities? |
| *Bodily pain (BP)* |
| How much bodily pain have you had? |
| How much did pain interfere with your normal work? |
| *Social functioning (SF)* |
| To what extent have your physical health or emotional problems interfered with your normal social activities with family, friends, neighbours, or groups? |
| How much of the time has your physical health or emotional problems interfered with your social activities? |
| *Role limitations due to emotional problems (RE)* |
| Did you cut down on the amount of time you spent on work or other activities? |
| Did you accomplished less than you would like? |
| Did you do work or other activities less carefully than usual? |
| *Vitality (VT)* |
| Did you feel full of pep? |
| Did you have a lot of energy? |
| Did you feel worn out? |
| Did you feel tired? |
| **SAQ7** |
| *Physical Functioning (PF)* |
| PF1: Walking indoors on ground level |
| PF2: Gardening, vacuuming or carrying groceries |
| PF3: Lifting or moving heavy objects (e.g., furniture, children) |
| *Angina Frequency (AF)* |
| AF1: I have had chest pain, chest tightness or angina |
| AF2: I have taken nitro-glycerine |
| *Quality of Life (QoL)* |
| QoL1: How much has your chest pain, chest tightness or angina limited your enjoyment in life? |
| QoL2: If you had to spend the rest of your life with your chest pain, chest tightness or angina the way it is right now, how would you feel about this? |

**Supplementary Material 1: In- and exclusion criteria**

Patients were eligible if they were 18 years or older, had stable CAD and were scheduled for elective coronary artery bypass graft (CABG) or elective percutaneous coronary intervention (PCI). Patients had to have at least one of the following somatic comorbidities: diabetes mellitus, obesity (body mass index > 30 kg/m2), joint disease (rheumatism, arthritis, osteoarthritis, or gout), pulmonary disease (asthma, chronic obstructive pulmonary disease, or bronchiectasis) or another chronic disease, e.g., psoriasis, hypertension, HIV, peripheral facial paresis. Patients with cognitive impairments due to brain haemorrhage, cerebral infarction, mental retardation, dementia, Alzheimer's disease, or patients who were unable to complete questionnaires due language problems were excluded.

**Supplementary Material 2, detailed results of the SEM procedure for the SF-36, Steps 1 and 2**

*Step 1*. Based on the manual of the SF-36 (Ware et al., 1993) we chose a two-factor model as a starting point for the measurement model, where physical health (PCS) is measured by PF, RP, BP, and GH, and mental health (MCS) by RE, VT, MH, SF (see Figure 1). The initial measurement model showed poor overall model fit (see Table 3, step 1). To arrive at a well-fitting measurement model we included three model-modifications. First, a residual covariance between item RP and RE was added (∆χ^2^ (4) = 38.777, *p* < .001). Adding this parameter means that RP and RE have something in common that is not explained by the underlying factors. Adding this parameter made sense given that both items measure one’s role in a social situation, a specific aspect that is not measured in the other items of the questionnaire. Second, a factor loading of VT on the factor PCS was added (∆χ^2^ (2) = 36.562, *p* < .001) which means that VT is not only indicative of the mental but also of the physical component of HRQoL. This modification is warranted because vitality and physical health are associated [38,39]. Finally, a factor loading of SF on the factor PCS was added (∆χ^2^ (2) = 31.569, *p* < .001), which means that social functioning is also indicative of the physical component, which could be justified theoretically as it is easier to engage in social activities when physically healthy [40]. With these modifications the measurement model showed reasonable and good overall model fit according to the RMSEA, CFI and SRMR, respectively. We therefore considered this to be the final measurement model (see Figure 1).

*Step 2*. In the no response shift model, all model parameters that are associated with response shift were set to be equal across measurements; i.e., imposing across measurement invariance for the factor loadings and intercepts. The no response shift model fitted significantly worse as compared to the measurement model (∆χ^2^ (14) = 25.894, *p* = .027), indicating the presence of overall response shift (see Table 3, step 2).
